# *ExtSpecR*: An R Package and Tool for Extracting Tree Spectra from UAV-Based Remote Sensing

**DOI:** 10.34133/plantphenomics.0103

**Published:** 2023-10-16

**Authors:** Zhuo Liu, Mahmoud Al-Sarayreh, Cong Xu, Federico Tomasetto, Yanjie Li

**Affiliations:** ^1^ State Key Laboratory of Tree Genetics and Breeding, Research Institute of Subtropical Forestry, Chinese Academy of Forestry, Hangzhou, Zhejiang 311400, China.; ^2^ Key Laboratory of Tree Breeding of Zhejiang Province, Research Institute of Subtropical Forestry, Chinese Academy of Forestry, Hangzhou, Zhejiang 311400, China.; ^3^ Department of Computer Engineering, German Jordanian University, Amman 11180, Jordan.; ^4^ School of Forestry, University of Canterbury, Private Bag 4800, Christchurch 8140, New Zealand.; ^5^ AgResearch Ltd., Christchurch 8140, New Zealand.

## Abstract

The development of unmanned aerial vehicle (UAV) remote sensing has been increasingly applied in forestry for high-throughput and rapid acquisition of tree phenomics traits for various research areas. However, the detection of individual trees and the extraction of their spectral data remain a challenge, often requiring manual annotation. Although several software-based solutions have been developed, they are far from being widely adopted. This paper presents *ExtSpecR*, an open-source tool for spectral extraction of a single tree in forestry with an easy-to-use interactive web application. *ExtSpecR* reduces the time required for single tree detection and annotation and simplifies the entire process of spectral and spatial feature extraction from UAV-based imagery. In addition, *ExtSpecR* provides several functionalities with interactive dashboards that allow users to maximize the quality of information extracted from UAV data. *ExtSpecR* can promote the practical use of UAV remote sensing data among forest ecology and tree breeding researchers and help them to further understand the relationships between tree growth and its physiological traits.

## Graphical abstract

**Figure:**
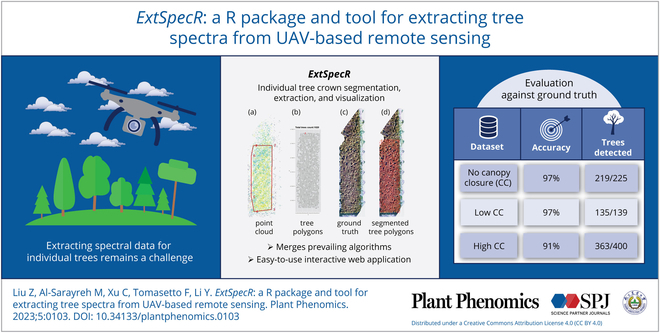

## Introduction

Forests are multifunctional systems; forest ecology and environmental policy, tree breeding, cultivation, and management are inseparable from forest inventory [1]. Forest inventory data have been used for a wide range of purposes, from basic quantification of timber productivity to assessment of forest biodiversity and carbon sequestration, as well as breeding of excellent tree germplasm resources and detailed analysis of forest resource distribution [2,3]. Therefore, forest inventory is the first and most important step in the study of forest ecology.

Unmanned aerial vehicles (UAVs), with the advantage of high-throughput data acquisition, have been widely used in forestry [4]. Advances in optical imaging sensor technologies and their application to UAV development have led to UAV-based remote sensing with RGB (red, green, and blue), Light Detection and Ranging (LiDAR), and multispectral/hyperspectral imaging [5]. These technologies are capable of increasing fieldwork efficiency with high accuracy and resolution [6]. The most common applications of UAV remote sensing in forestry are focused on species classification [7], individual tree detection [8], insect pest control [9], and breeding selection [10].

Individual tree detection and segmentation are critical workflow steps for UAV remote sensing to achieve accurate results when analyzing forest scenes. To achieve this, the point cloud generated by LiDAR and imaging sensors using structure-from-motion (SfM) technology can be used to extract relative information of each tree, such as RGB, multispectral/hyperspectral, and distance to the ground. The extracted information is then combined with segmentation algorithms, including marker-controlled watershed segmentation [11,12], seeded region growing [13], Voronoi tessellation [14], and point cloud-based cluster segmentation [15]. The accuracy of the extracted information for individual trees substantially affects the overall accuracy of forest analysis tasks, such as tree classification or phenotypic trait prediction [16].

In addition, advanced object detection models based on convolutional neural networks (CNNs) can be used for individual tree localization (tree detection task) and classification (tree recognition task). Widely used region-based CNN (R-CNN) models, such as faster R-CNN and mask R-CNN, have achieved successful tree detection and segmentation based on either UAV multispectral [17] or RGB imagery [18]. R-CNN models analyze the image and extract self-features based on a predefined ground truth of each tree in the image. Therefore, CNN-based approaches show better performance for tree detection and classification than traditional segmentation algorithms [19]. However, it should be noted that these approaches require manual labeling (annotation) to generate the ground truth for each tree in the image, and the accuracy of the annotation process affects the overall accuracy of the models used to train the R-CNN model.

In practice, an R package called lidR [20] provides an implementation of several segmentation and clustering algorithms that can be used for point cloud analysis and individual tree segmentation. The lidR package consists of several functions for handling point clouds with high-precision positioning. To extract the associated spectral information of each tree, the process in the lidR package starts by classifying the point cloud data as either ground or non-ground points, which can be used to create the Digital Terrain Model (DTM) and then the Digital Surface Model (DSM). The DSM and DTM are then used to compute canopy height models, which can be used to segment individual trees and generate an individual canopy polygon for each tree. Finally, the individual tree crown polygons are used to extract multispectral information from the images using another R package called raster [21].

For forest ecologists and tree breeders, it is important to accurately and quickly locate each tree in a large forest plot to understand its growth situation and tree distribution, and to study tree species diversity. Manual labeling of each tree with low throughput is time-consuming and labor-intensive for large-scale or multitemporal data. Therefore, a high-throughput method to extract spectral information from a single tree is extremely useful. Thus, the main objectives and contributions of this paper and the developed tool *ExtSpecR* are the following:

1. Presentation and guidelines: This paper offers a comprehensive overview and detailed guidelines for the *ExtSpecR* tool, elucidating its structure, features, functionalities, and illustrative examples that underscore the tool's robustness in real-world applications.

2. User-centric design: The *ExtSpecR* tool is equipped with a user-friendly graphical user interface (GUI) designed for efficient spectral extraction and visualization of individual trees in remotely sensed imagery. It caters especially to forest ecologists and tree breeders, including those without a programming background.

3. Innovation in spectral extraction: *ExtSpecR* emerges as one of the pioneering free and open-source tools, uniquely integrating a plethora of features to autonomously extract spectra from individual trees in remotely sensed imagery.

4. Efficiency and accessibility: *ExtSpecR* boasts flexible input and output routines that expedite the spectral extraction process. This efficiency paves the way for swift forest inventories, broadening the reach of UAV technologies to a wider audience, including non-experts.

## Materials and Methods

### General workflow

*ExtSpecR* is a comprehensive tool designed to provide intuitive methods for analyzing and visualizing tree attributes from various remote sensing data sources. Prioritizing user-friendliness, the tool ensures that even those unfamiliar with complex remote sensing methodologies can easily access its capabilities. The workflow of *ExtSpecR* can be divided into 2 primary procedures:

#### 2D data processing: Visualization of multispectral images

**Fig. 1.**
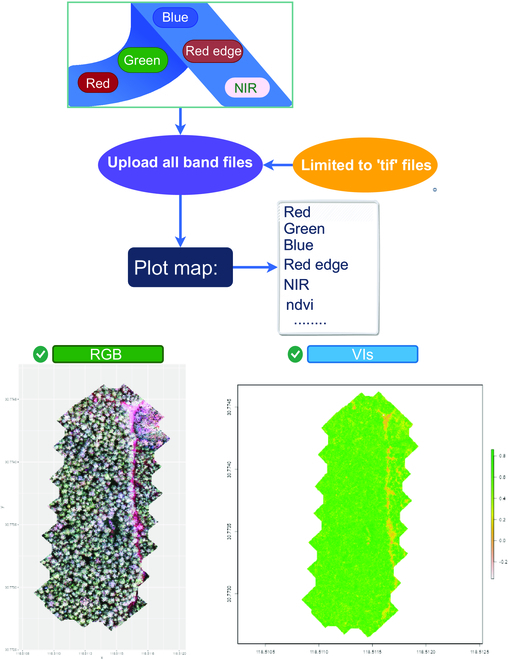
The workflow for the VIs generation process that is implemented in VIs generation. The input channels can be for an individual tree or scene as shown in this example.

*ExtSpecR* facilitates a clear understanding of forest characteristics across different light spectra. The multispectral images reveal various forest attributes such as vegetation health, tree species, and potential hazards within the forest ecosystem. Figure 1 presents a simplified procedure, depicting the transformation from raw image data into informative visuals.

**Table.** The size of the point cloud and raster images and time spent to do the forest inventory (tree identification, segmentation, and spectral extraction) using the *ExtSpecR* tool.

Data type	Point cloud				Total raster image size (MB)	ROI segmentation time (min)	ROI extraction time (min)	Total tree count	(Manually label) Ground truth	Detected trees	Accuracy
	Size (MB)	Density (points/m^2^)	Points (million)	Area (km^2^)							
No CC	28.8	1.9	1.16	0.61	370	4. 45	68.23	3,817	225	219	97%
Low CC	61.3	1.34	16.6	0.16	470	0.69	22.07	1,020	139	135	97%
High CC	1,240	62.9	51.34	0.82	1,270	13.2	302.48	18,463	400	363	91%

#### 3D data processing: Comprehensive analysis using LiDAR point clouds and multispectral data

**Fig. 2.**
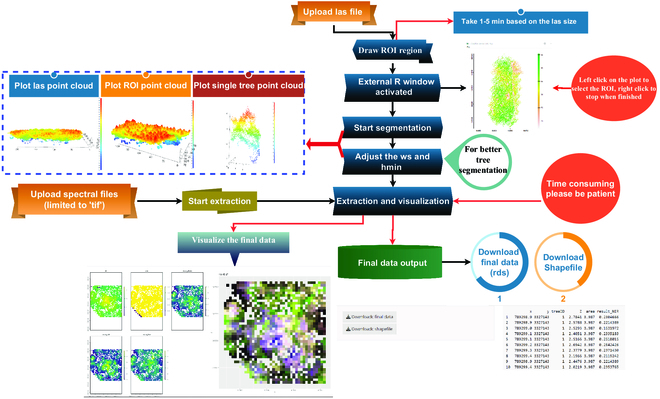
The workflow for the data process and dowload submenu in tree phenotyping.

*ExtSpecR* integrates the depth of 3-dimensional (3D) point clouds with multispectral data for a comprehensive analysis of individual trees and the broader forest ecology. Initially, point clouds data are processed to extract tree attributes like height, crown area, and other structural details. After defining the regions of interest (ROIs) or individual tree crown regions, *ExtSpecR* extracts the spectral information from the provided multispectral images as well as the tree height and crown area from the point clouds, ensuring the data correspond directly to the individual tree. This method results in a dataset that combines both structural and spectral information for each tree. Figure 2 visually represents the entire workflow.

### Algorithm and implementation

#### Data input: Uploading and visualizing spectral images

In the VIs Graphs Generation stage, users initiate the data input process by uploading a spectral image, which could be from a single tree or an entire scene. The tool accepts up to 5 channels: red, green, blue, red edge, and near-infrared spectroscopy (NIR). It is important to emphasize that the tool only processes images in the TIFF format.

#### VI calculation, individual tree segmentation, and traits calculation

Upon accessing the phenotyping menu, users are introduced to 2 primary sub-panels: “Data Process” and “Download”. The process begins with the upload of point cloud data, either in “las” or “laz” format, and the associated multispectral image. Users then define an ROI for focused analysis. Following this, the segmentation process is initiated based on user-defined parameters, leveraging the “locate_trees” function from the lidR package. Once segmentation is complete, *ExtSpecR* provides a 3D view and detailed spectral information of the segmented trees.

#### Visualization and data output: Reviewing and downloading data

After processing, *ExtSpecR* presents users with a comprehensive interface to review segmented trees and their spectral data. The tool also offers downloading options, with the data available in both standard and shapefile formats for compatibility with GIS applications.

#### Dependencies

The lidR Package: *ExtSpecR* heavily relies on the lidR package [20,22] for functionalities like individual tree identification and tree segmentation. This package provides the foundational algorithms that *ExtSpecR* builds upon. However, *ExtSpecR*'s unique contribution is its user-friendly user interface (UI) wrapper, making these advanced functionalities accessible to non-expert users.

While lidR is a core dependency, *ExtSpecR* also utilizes other R packages for a range of functionalities, from data manipulation to spectral extraction. Each of these packages contributes to the holistic functioning of *ExtSpecR*, ensuring comprehensive data analysis for users. For a detailed list of dependencies and their specific roles within *ExtSpecR*, users can refer to the GitHub repository: https://github.com/Yanjie-Li/ExtSpecR.

#### Implementation details

The *ExtSpecR* package is hosted on GitHub and is licensed under Massachusetts Institute of Technology (MIT). The repository ensures ease of access and provides comprehensive information for installation. Moreover, it includes a tutorial with sample data for hands-on analysis. Recognizing the diverse user base, a portable version is also available for those not acquainted with R. For optimal performance, users should ensure they have the latest versions of R and all dependency packages. The tool has been tested in a robust personal computer environment, specifically on a system running Windows with R version 4.2.3 (2023-03-15), equipped with 192 GB of memory, and an NVIDIA GeForce RTX 2080 Ti graphics processing unit (GPU) with 11.0 GB of GPU memory, ensuring smooth operation and optimal performance.

## Results

### Functional results: User interface and outputs

**Fig. 3.**
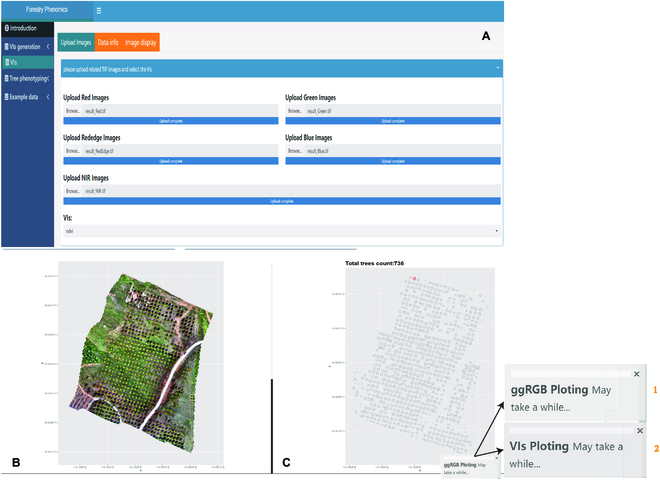
The primary components and functions of the VIs Graphs Generation page. (A) The input buttons and control panel used to select the plot type and plot saving options. (B and C) The ontology panel illustrating an example of a false-color image and VIs graph generated from a multispectral image of a remote sensing scene.

The *ExtSpecR* tool provides an integrated approach for vegetation index (VI) calculation and visualization. As depicted in Fig. 3A, the platform supports TIFF formatted spectral image uploads, accommodating up to 5 distinct channels, namely red, green, blue, red edge, and NIR. Once the necessary channels are uploaded, users are endowed with the flexibility to specify desired VIs for computation. Subsequently, the system generates 2 distinct visual outputs: a false-color representation of the input data (Fig. 3B) and a VI-specific image (Fig. 3C). The provision to download these outputs in TIFF format further accentuates the tool’s user-centric design.

**Fig. 4.**
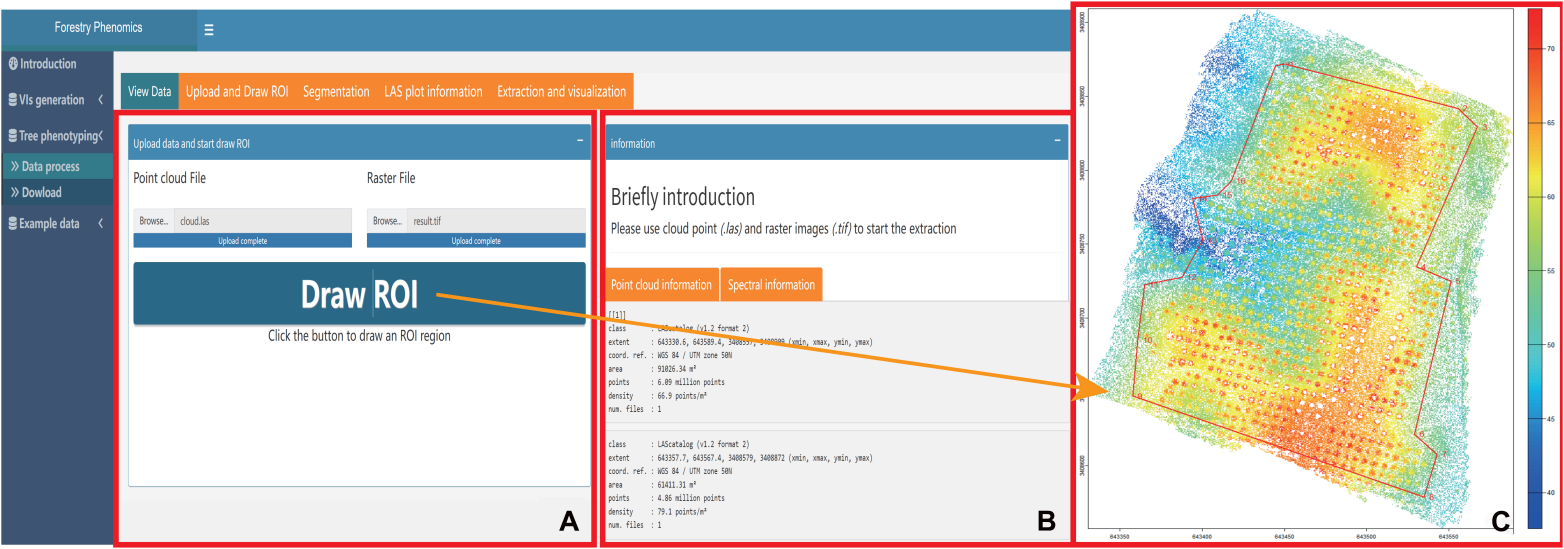
The panel of Upload and Draw ROI page. (A) The upload area and the “Draw ROI polygon” button. (B) The information panel about the point cloud and spectral information; the external panel after click on the “Draw ROI polygon” button. (C) An example of a drawn ROI region in a point cloud of data.

Transitioning to the core phenotyping capabilities of *ExtSpecR*, Fig. 4 delineates its structured interface, bifurcated into “Data Process” and “Download” sub-panels. Under the “Data Process” sub-panel, a sequential workflow is initiated with the “Upload and Draw ROI” page. Herein, users are required to upload their point cloud data (either “las” or “laz” formats) and the corresponding multispectral image (with channels represented in TIFF format, as illustrated in Fig. 4A). Upon successful data ingestion, the interface provides immediate visual feedback by displaying the spectral and point cloud data (Fig. 4B).

A salient feature embedded within this workflow is the “Draw ROI polygon” tool. Activation of this tool initiates an external R graphics window, as demonstrated in Fig. 4C. Within this environment, users are afforded the capability to manually demarcate their ROI, foundational for subsequent tree identification and segmentation. To ensure precision and user satisfaction, *ExtSpecR* allows iterative adjustments of the ROI.

**Fig. 5.**
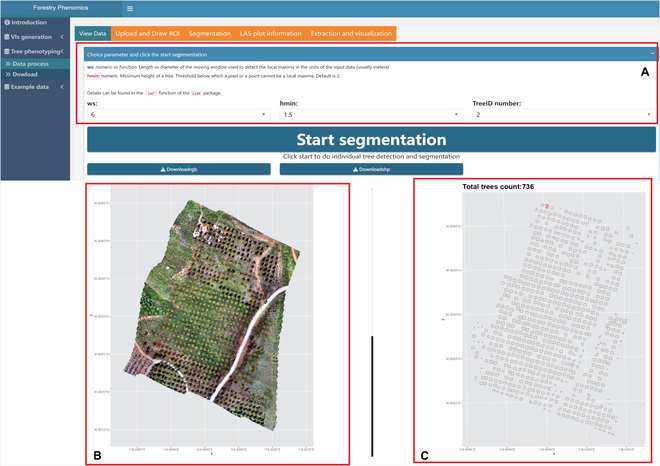
The interface of the “Tree identification and Spectral extraction” page. (A) Customization and control panel. (B) Ontology panel. (C) Information panel.

**Fig. 6.**
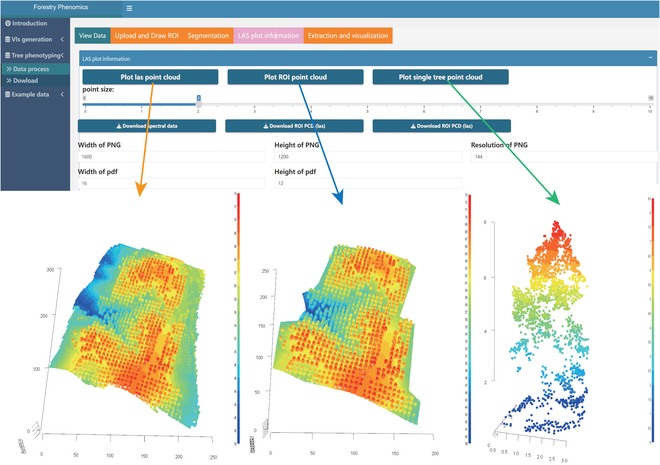
The point cloud and spectral information panels.

Post-ROI specification, users transition to the segmentation phase. Here, parameters such as window size and minimum height can be defined by the user. The underlying segmentation mechanism leverages the “locate_trees” function from the lidR package. The culmination of the segmentation process is visually presented to the user. In instances where multiple images are provided, a false-color image representation is generated, as portrayed in Fig. 5. For a granular spatial understanding, the tool offers a 3D visualization of the segmented trees, rendered using the rgl package (Fig. 6).

**Fig. 7.**
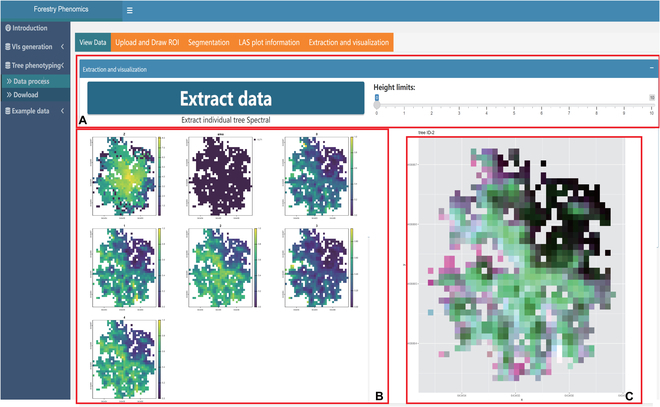
The extraction and visualization panel.

**Fig. 8.**
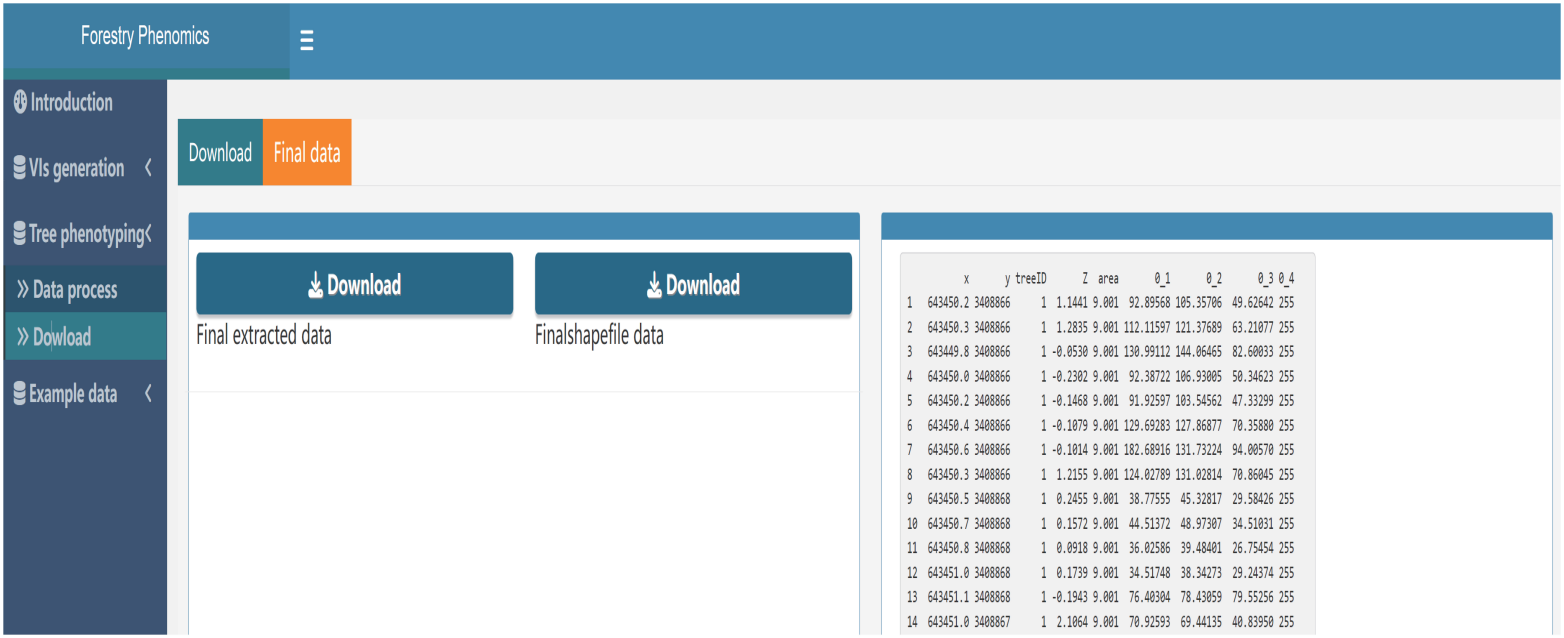
The final downloadable data output panel and format.

The final phase encapsulates spectral information extraction from the delineated trees. The “Extraction and Visualization” panel facilitates this, where users can dynamically view spectral data based on tree heights, a feature exemplified in Fig. 7. To enhance data accessibility, the platform not only provides visualization but also allows data retrieval. Comprehensive datasets, encompassing metrics such as longitude, latitude, tree ID, point height, canopy area, and spectral data, can be effortlessly downloaded, as illustrated in Fig. 8.

### Feasibility/evaluation results: Comparison with ground truth and evaluation metrics

In the quest to substantiate the accuracy and functionality of the *ExtSpecR* tool, it is imperative to cross-verify its outputs against known standards or “ground truths”. The following section amalgamates the findings from the “Example Data” and “Example Applications” sections to present a cohesive evaluation of the *ExtSpecR* tool.

#### Sample data presentation

**Fig. 9.**
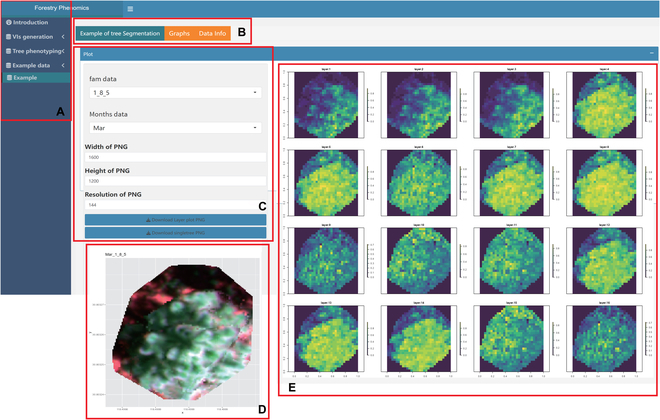
The interface of the segmentation page of *ExtSpecR_app.* (A) Navigation panel. (B) Customization and control panel. (C and D) Ontology panel. (E) Information panel.

To elucidate the data structures and visual outputs generated by *ExtSpecR*, a sample data menu has been curated. This menu, distinctively structured around data rather than functions, furnishes users with a clear perspective of the spectral and spatial information extraction capabilities of individual trees. The sample datasets are meticulously organized based on 2 criteria: family (fam) and month. The interface, as showcased in Fig. 9, facilitates seamless navigation through the spectral and VI channels (Fig. 9C) and also provides a false-color representation of the analyzed tree (Fig. 9D). Users are endowed with the flexibility to select specific trees based on the “fam” and “month” criteria using a dropdown menu (Fig. 9B). Moreover, the platform provides intricate details via the Image Info and Data panels, ensuring users are well-informed. The tool also offers customization capabilities, allowing users to adjust the plot dimensions and resolutions of individual trees and subsequently download them (Fig. 9C).

#### Evaluation against ground truth

**Fig. 10.**
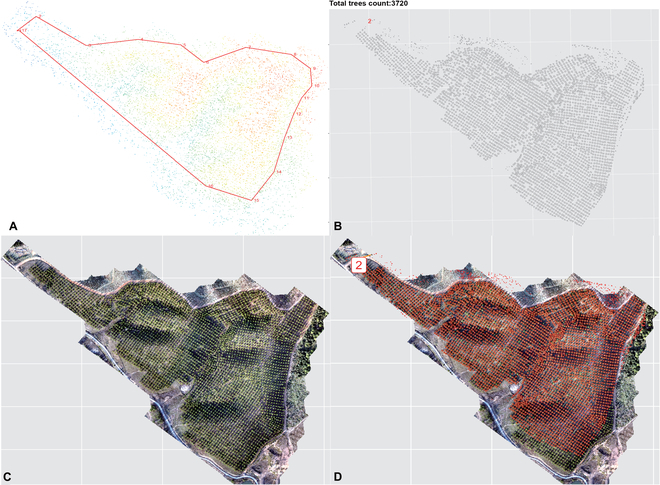
Tree information extraction on a large-scale tree plantation with no crown closure. (A) ROI selected area from the point cloud. (B) Segmented trees polygon. (C) Ground truth RGB images. (D) Display of segmented tree polygons on the ground truth image.

**Fig. 11.**
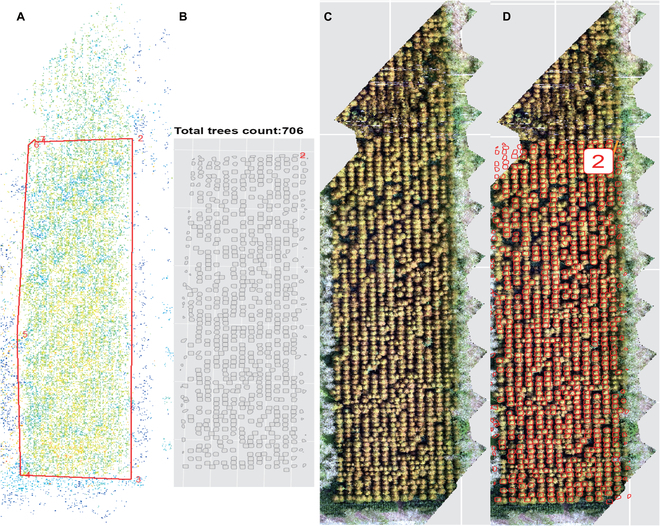
Tree information extraction on a tree plantation with low crown closure. (A) ROI selected area from the point cloud. (B) Segmented trees polygon. (C) Ground truth RGB images. (D) Display of segmented tree polygons on the ground truth image.

**Fig. 12.**
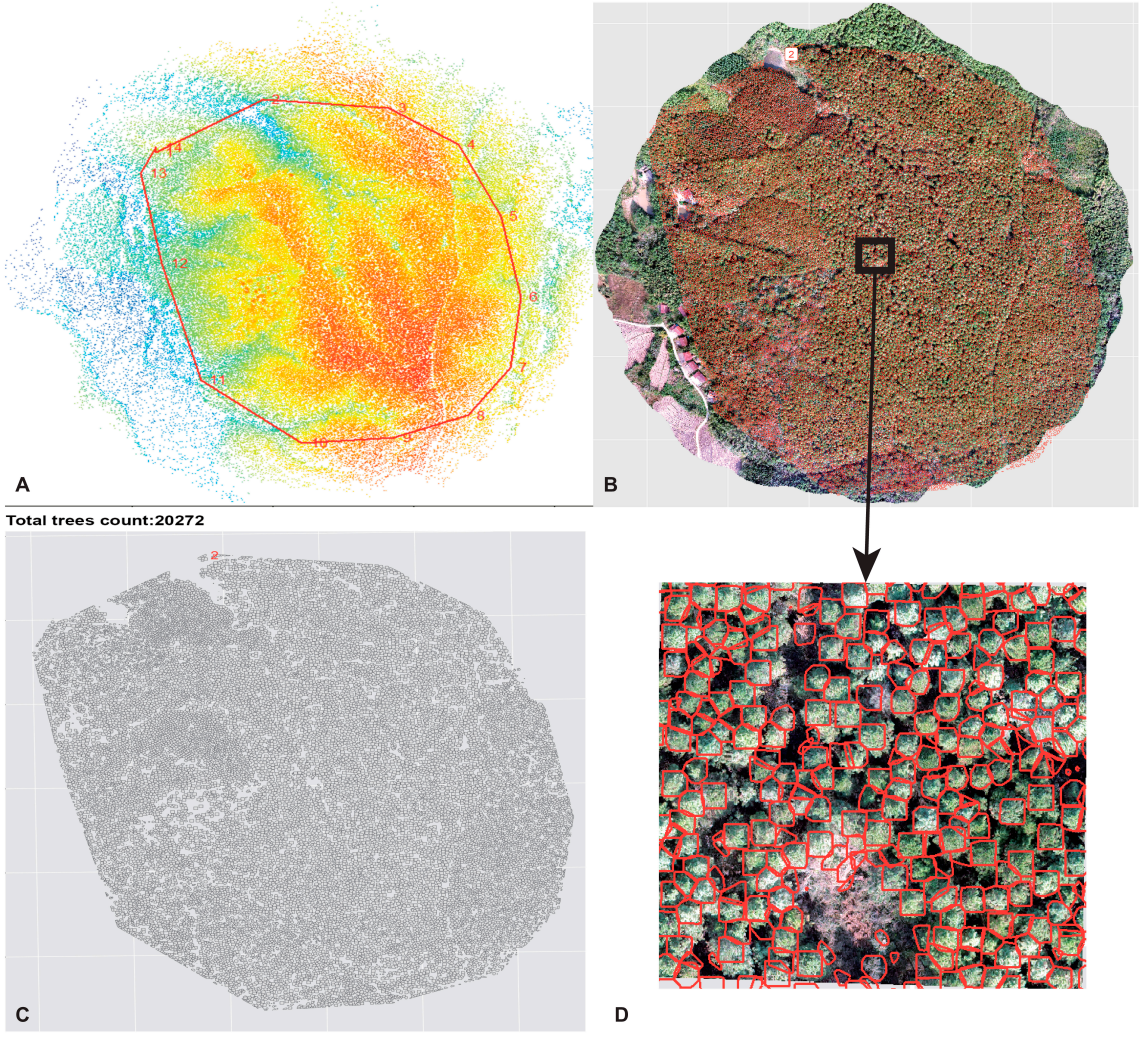
Tree information extraction on a complex tree plantation with high crown closure. (A) ROI selected area from the point cloud. (B) Segmented trees polygon. (C) Display of segmented tree polygons on the ground truth image. (D) Zoom in on a randomly selected region.

The *ExtSpecR* tool was extensively tested on 3 different types of tree plantations with varying canopy closure (CC) densities (Figs. 10 to 12) to assess its resilience and ability to segment individual tree crowns at different canopy closure levels. Each dataset was processed using DJI Terra (version 3.4.4, Shenzhen, China) and included point cloud files and multispectral images, encompassing 5 bands in TIFF format. The “no CC” dataset, which lacked significant canopy interference, resulted in simpler data processing. Table displays the results, detailing a point density of 1.9 points/m^2^ spread over an area of 0.61 km^2^. The dataset proved to be 97% accurate, identifying 219 trees from a ground truth of 225. The processing time of ROI segmentation took approximately 4.45 min, which was inherently influenced by the simplicity of the dataset. The “low CC” dataset was well-balanced, providing insightful tree details without excessive complexity. This dataset demonstrated a density of 1.34 points/m^2^ across an area of 0.16 km^2^ and exhibited a commendable 97% accuracy, successfully detecting 135 trees relative to a manually annotated count of 139. The more complex “high CC” dataset presented challenges due to the high overlap rate of the canopy layers, making individual tree identification particularly challenging. This dataset was dense, with 62.9 points/m^2^ covering an area of 0.82 km^2^. From a ground truth count of 400 trees, the dataset managed to detect 363 trees, resulting in an accuracy of 91%. Due to its complexity, the processing times for this dataset were notably extensive, taking up to 302.48 min for extraction.

## Discussion

The primary objective of the *ExtSpecR* tool is to identify trees and acquire their spectral spatial data for subsequent use. The primary issue lies in the large size of the input point cloud and spectral images. The tool has a maximum upload file size limit of 10 GB with an upload duration of about 6 min. As the amount of data increases, both the upload time and data processing time significantly increase. To reduce processing time, we recommend that users segment their point cloud data and upload only the regions of interest. Additionally, detection is complicated in complex environments where tree canopies overlap. In our testing of diverse forest types, the tool demonstrated notable performance, even in areas with substantial tree overlap. To reduce the impact of nearby vegetation, we propose defining the specific target area, as this approach yielded more dependable outcomes. There are also some other free and open-source tools available for comparable objectives, including the following: FSCT: https://github.com/SKrisanski/FSCT, TreeTool: https://github.com/porteratzo/TreeTool, and Forest 3D App: https://github.com/lloydwindrim/forest_3d_app.

While *ExtSpecR* shares some functionalities with other tools, it stands out due to its seamless integration, intuitive visualizations, and user-friendly interface. We do not claim that *ExtSpecR* is unprecedented, but rather highlight its unique strategy of utilizing and merging prevailing algorithms to deliver an optimized user experience. FSCT offers strong capabilities for segmentation, but its main focus is on individual tree crown segmentation, extraction, and visualization. TreeTool is also an option to consider. This software provides several functionalities for processing tree-related data. *ExtSpecR* offers an integrated workflow that combines point cloud data with multispectral imaging for comprehensive tree analysis. Forest 3D App primarily focuses on presenting spatial representations of forest plots while *ExtSpecR* supplements this visualization with in-depth spectral data extraction and analysis.

Further enhancements for *ExtSpecR* include improving cloud quality. We are currently developing an application that uses the SfM algorithm to detect trees and extract their spectral information from UAV-based imagery. Currently, there has been no evaluation of this application’s efficiency with LiDAR point clouds. However, testing with hyperspectral images has the potential to enhance the tool’s capacity to extract hyperspectral data for individual trees.

In summary, we have introduced *ExtSpecR*, an open-source R package and tool developed to find the tree position and extract the tree spectral information using UAV-based point clouds and multispectral imagery. *ExtSpecR* employs a user-friendly interactive GUI to minimize the effort required by the user and to maximize the quality of the extracted information. *ExtSpecR* demonstrates that the process of automatically generating tree positions can be optimized and the process can be faster than using various functions to perform the extraction process. We expect that *ExtSpecR* will be a useful tool to accelerate and simplify plant phenomics extraction processes.

## Data Availability

The *ExtSpecR* package is available in a GitHub repository (https://github.com/Yanjie-Li/ExtSpecR) under the MIT license.
